# Supplementary material for: Transcriptomic analysis links gene expression to unilateral pollen-pistil reproductive barriers
Source: BMC Plant Biol. 2017 Apr 24;17:81. doi: 10.1186/s12870-017-1032-4 (PMC5402651; doi:10.1186/s12870-017-1032-4)
Supplement: Supplementary file 2 — This PDF contains all of the additional material (Figure S1, Figure S2 and Method S1) associated with the manuscript. Figure/Method numbers and titles are listed below. Figure S1. The genome-wide patterns of expression in styles from two populations of Solanum habrochaites. Figure S2. Sequence alignments of hypothetical proteins showing the highest fold-change in UI-competent vs. UI-compromised tissues. Method S1 Full command and parameters used for SHEAR in bioinformatics analysis of Solanum habrochaites transcriptomes. (DOCX 52 kb) [file 12870_2017_1032_MOESM2_ESM.docx]

**Linking gene expression to unilateral pollen-pistil reproductive barriers**

Amanda K. Broz^1,*^, Rafael F. Guerrero^2,*^, April M. Randle^1,3^, You S. Baek^1^, Matthew W. Hahn^2,4^, Patricia A. Bedinger^1^

**Additional File 2.**

**Figure S1.** The genome-wide patterns of expression in styles from two populations of *Solanum habrochaites* (LA1777 in orange and LA0407 in green), summarized by a principal components analysis. Most variance among samples is due to differences between populations (horizontal axis), and we found no large differences among experimental treatments at this scale (unpollinated styles, stars; self-pollinated, circles; intrapopulation-pollinated, triangles; interpopulation pollinated, squares).

**A**

Penn MALVSWAKKELSKLKLQNKPKRLTLPQTSTK**CLAL**PLIQEVILDADLRCTHCQNRVSSVI

Lyco ---VSWAKKELSKLKLQNKPKRLTLPQTSTK**CLAL**PLIQEVILDADLRCTHCQNRVSSVI

*********************************************************

Penn SNVEDVESIVVHVLEKKVTLIRKSTSK

Lyco SNIEDVESIVVHVLEKKVTLIRKSTSK

**:************************

**B**

Penn **MAGRVMLGVCVIFFVVASVAS**ITPAPSPNVAESPVDNNVIGTLDGGVGGAAPVGGPVPEG

Lyco **MAGHVMLGVCVIFFVVASVAS**ITPAPSPNVAESPVDNNVIGTLDGGVGGAAPVGGPVPEG

***:********************************************************

Penn VFSNISPESESSAATINAHLSTIAIISSIVATSFLLS

Lyco VFSNISPESQSSAATINAHLSTIAIISSIVATSFLLS

*********:***************************

**Figure S2.** Sequence alignments of hypothetical proteins showing the highest fold-change in UI-competent vs. UI-compromised tissues. **A,** The deduced amino acid sequence of *Solanum pennellii* Sopen12g017530 (Penn), the gene showing the highest fold change in UI-competent versus UI-compromised styles, is aligned to a *Solanum lycopersicum* (Lyco) EST (GenBank # AW092656.1) identified in an elicitor screen of tomato leaf. A BLAST search of *S. lycopersicum* gene models (SOLv2.4, solgenomics.net) did not return any results. Putative heavy metal binding domain (P-Fam 00403) is shown in blue; putative prenylation site (CaaX, where ‘a’ represents an aliphatic amino acid) is shown in bold “CLAL”. Cysteine residues involved in both domains are underlined. Stars represent residues conserved between both sequences. **B,** The deduced amino acid sequence of *Solanum pennellii* Sopen12g014190 (Penn), the gene showing the highest fold change in UI-competent versus UI-compromised pollen, is aligned with *Solanum lycopersicum* Solyc12g033100 (Lyco). Putative signal peptide is shown in bold; distinguishing dipeptide motifs of arabinogalactan proteins (AGPs) are underlined. Hydrophobic residues are highlighted in yellow. Stars represent residues conserved between both sequences.

**Method S1.**  Full command and parameters used for SHEAR in bioinformatics analysis of *S. habrochaites* transcriptomes.

#!/bin/bash

# These script snippets were used for the differential gene expression analysis presented in

# Broz et al. "Transcriptomic characterization of a pollen-pistil unilateral reproductive barrier"

# These will not run adequately without helper files (as indicated below)

# Software needed:

# scythe (github.com/vsbuffalo/scythe)

# shear (github.com/jbpease/shear)

# subread (subread.sourceforge.net/)

# STAR (github.com/alexdobin/STAR/)

# samtools (www.htslib.org/)

# Contact: Rafael F Guerrero, rafguerr@indiana.edu

# (1) Shear reads

#raw_R1_fullpaths.txt must contain all the names of the R1 fastq files to be analyzed

while read line

do

a=${line:0:${#1}-9}

python shear.py \

--fq1 $a"_R1.fastq" \

--fq2 $a"_R2.fastq" \

--out1 $a"_sheared_R1.fastq" \

--out2 $a"_sheared_R2.fastq" \

--execscythe /N/soft/rhel6/scythe/0.992beta/scythe \

--tempdir $(pwd)/tempfiles_shear \

--trimfixed 0:0 --trimqual 20:20 --trimqualpad 0:0 --filterlength 50 --trimpattern3 AGATC --trimpolyat 12 --trimambig --filterlowinfo 0.5 --filterunpaired --filterqual 10 --filterambig 8

done < raw_R1_fullpaths.txt

# (2) STAR mapping

#shear_R1_fullpaths.txt must contain all the names of the R1 fastq files preprocessed by shear

# The variable PATH_TO_GENOME must be set to the absolute path to the (STAR indexed) reference genome

while read line

do

prefix=${line:0:${#line}-17}

dirname=${prefix}_penn

if [ ! -d "$dirname" ]; then

mkdir $dirname

cd $dirname

STAR \

--genomeDir $PATH_TO_GENOME \

--readFilesIn ${prefix}_sheared_R1.fastq /${prefix}_sheared_R2.fastq \

--runThreadN 8 \

--outReadsUnmapped Fastx \

--genomeLoad LoadAndKeep

fi

done < shear_R1_fullpaths.txt

# (3) Sorting, filtering and indexing alignment files

#dirnames.txt must have the absolute paths to the STAR output directories (one per library)

while read line

do

cd $line

prefix="Aligned.out"

if [ ! -f ${prefix}".sorted.bam" ]; then

samtools view -uS ${prefix}".sam" > ${prefix}".bam"

samtools sort $prefix".bam" $prefix".sorted"

samtools index $prefix".sorted.bam"

rm $prefix".bam"

fi

done < dirnames.txt

# (4) Counting reads in gene models with featureCounts from subread-1.4.6

featureCounts -T 15 -B -p -t exon -g Parent -a spenn_v2.0_exons.gff -o all_counts_to_PENN.txt $(cat all_penn_bams.txt)
